# *In vitro *antimicrobial effects of aztreonam, colistin, and the 3-drug combination of aztreonam, ceftazidime and amikacin on metallo-*β*-lactamase-producing *Pseudomonas aeruginosa*

**DOI:** 10.1186/1471-2334-9-123

**Published:** 2009-08-10

**Authors:** Shigeharu Oie, Yumi Fukui, Masaya Yamamoto, Yuki Masuda, Akira Kamiya

**Affiliations:** 1 Department of Pharmacy, Yamaguchi University Hospital, Ube, Japan

## Abstract

**Background:**

There are limited choice of antimicrobial agents to treat infection with metallo-*β*-lactamase-producing *Pseudomonas aeruginosa*. We evaluate the antimicrobial effects of aztreonam alone, colistin alone and the 3-drug combination of aztreonam, ceftazidime and amikacin on 23 strains of metallo-*β*-lactamase-producing *P. aeruginosa *by time-killing tests.

**Methods:**

Strains used were from different hospitals in Japan and had different pulse-field gel electrophoresis patterns by restriction with *Spe*I. The minimum inhibitory concentrations of 11 antimicrobial agents (piperacillin, piperacillin/tazobactam, imipenem, meropenem, aztreonam, ceftazidime, amikacin, tobramycin, arbekacin, ciprofloxacin and colistin) were determined using the agar dilution test. The effects of aztreonam, colistin and the combination of aztreonam, ceftazidime and amikacin were determined by time-killing studies.

**Results:**

Bacteriostatic effects after 6 hours of drug exposure were observed in 12 strains (52.2%) of 23 strains of metallo-*β*-lactamase-producing *P. aeruginosa *with 48 mg/l aztreonam, in 19 strains (82.6%) with the 3-drug combination of 16 mg/l aztreonam, 16 mg/l ceftazidime, and 4 mg/l amikacin, and in 23 strains (100%) with 2 mg/l colistin. Bactericidal effects after 6 h drug exposure were observed in 1 strain (4.3%) with 48 mg/l aztreonam, in 8 strains (30.4%) with the 3-drug combination and in all 23 strains (100%) with 2 mg/l colistin.

**Conclusion:**

Evaluation of *in vitro *antimicrobial effects on metallo-*β*-lactamase-producing *P. aeruginosa *revealed relatively good effects of the 3-drug combination of aztreonam, ceftazidime and amikacin and marked effects of colistin.

## Background

*Pseudomonas aeruginosa *is a major bacterium causing nosocomial infection, and the development of multidrug resistance has become a problem [1-7]. Since metallo-*β*-lactamase (MBL)-producing *P. aeruginosa *is often resistant not only to all *β*-lactams, but also aminoglycosides, and fluoroquinolones, there are often no drugs to treat infection with this bacterium [8-10]. In addition, no extended survey involving a series of human infections with MBL-positive isolates has been performed to determine the optimal treatment. Thus, appropriate therapy for those infections remains unknown [11].

We previously reported the effects of the 3-drug combinations of aztreonam, ceftazidime and amikacin or aztreonam, piperacillin and amikacin on 7 strains of multidrug-resistant *P. aeruginosa *[8-10]. In this study, to confirm the effectiveness of the 3-drug combinations, we evaluated the effects on a total of 23 strains of MBL-producing *P. aeruginosa *isolated in 23 hospitals in Japan in comparison with the effects of aztreonam or colistin alone.

## Methods

### Bacterial strains

Among *P. aeruginosa *strains sent from hospitals in Japan to the Japanese National Institute of Infectious Disease for detailed examination between January 2007 and July 2008, MBL-producing *P. aeruginosa *strains were screened, and MBL typing was performed according to the method of Shibata *et al *[12]. All 23 strains (one strain/hospital) of MBL-producing *P. aeruginosa *isolated during this period were donated by the Department of Bacterial Pathogenesis and Infection Control, National Institute of Infectious Disease, and used for the experiments.

### Pulsed-field gel electrophoresis

The high-molecular-weight chromosomal DNA was prepared according to the method of Murray *et al *[13], and the DNA sample in a small slice of an agarose plug in 200 μl of reaction buffer was digested with 30 U *Spe*I (New England Bio Labs, USA). Pulsed-field gel electrophoresis was carried out with the Bio-Rad Gene Path system (Bio-Rad, USA) in a 1% agarose gel in 0.5 × TBE buffer at 14°C with a linear ramp time of 1 to 23 s over a period of 18.5 h. Thereafter, the gels were stained with ethidium bromide and photographed.

### Susceptibility tests using agar dilution methods

The minimum inhibitory concentrations (MICs) were determined after 18 h of incubation at 37°C by dilution on Sensitivity Disc Agar-N (Nissui Pharmaceuticals, Tokyo, Japan). The following antimicrobial agents were tested: piperacillin, piperacillin/tazobactam (Toyama Chemicals, Tokyo, Japan), imipenem, amikacin (Banyu Pharmaceuticals, Tokyo, Japan), meropenem (Dainippon-Sumitomo Pharmaceuticals, Tokyo, Japan), aztreonam (Eisai Co., Tokyo, Japan), ceftazidime (Glaxo Japan Co., Tokyo, Japan), tobramycin (Shionogi Pharmaceuticals, Tokyo, Japan), arbekacin (Meiji Seika Co., Tokyo, Japan), ciprofloxacin (Bayer Japan Co., Tokyo, Japan) and colistin (Wako Junyaku Co., Osaka, Japan). These antibiotics except for ciprofloxacin were provided in the form of a freeze-dried amorphous powder. The inocula (10^4 ^colony-forming units [cfu]/spot) were plated using a multipoint inoculator (Sakuma Co., Tokyo, Japan). The MIC was defined as the lowest drug concentration that inhibited visible growth. *P. aeruginosa *IFO 3919 was used as the reference strain. The drug concentrations (breakpoints) were set as follows: piperacillin, 64 mg/l; imipenem and meropenem, 8 mg/l; aztreonam and ceftazidime, 16 mg/l; amikacin, tobramycin and arbekacin, 4 mg/l; ciprofloxacin and colistin, 2 mg/l. Breakpoints used for all agents (except for amikacin, tobramycin, arbekacin and colistin) were according to the National Committee for Clinical Laboratory Standards (NCCLS) criteria [14]. The concentration of amikacin and tobramycin was 4 mg/l, which is lower than the criteria of the NCCLS. This was because in Japan, the routine dose of these agents is lower (ex. in the case of amikacin, 200–400 mg/day in one to two divided doses) than that in Western countries. The concentration of colistin used was according to a report by Soussy *et al *[15].

### Drug effects in killing tests

Killing experiments were carried out to evaluate the bactericidal activities of 48 mg/l aztreonam, 2 mg/l colistin, and the 3-drug combination of 16 mg/l aztreonam, 16 mg/l ceftazidime and 4 mg/l amikacin. The final concentration of the log-phase inocula was approximately 10^5^–10^7 ^cfu/ml [16-19]. Viability was determined based on bacterial counts at 2, 4, 6 and 24 h after incubation with drugs at 37°C by plating 500 μl of serial dilutions from each tube onto trypticase soy agar plates followed by incubation of the plates at 37°C for 24 to 48 h. In a preliminary experiment, drug carryover was ruled out by plating samples of a bacterial suspension containing 2 × 10^2^– 4 × 10^2 ^cfu/ml in the presence or absence of antimicrobial agents alone or in combination. We also carried out preliminary killing tests with the 3-drug combination of 16 mg/l aztreonam, 16 mg/l ceftazidime and 4 mg/l amikacin, of 16 mg/l aztreonam, 64 mg/l piperacillin and 4 mg/l amikacin, and of 16 mg/l aztreonam, 64 mg/l piperacillin/4 mg/l tazobactam and 4 mg/l amikacin on 23 strains of MBL-producing *P. aeruginosa*. As a result, the viable cell count at 4 h after drug addition decreased to 1/100 or less of the initial count in 12 strains with aztreonam, ceftazidime and amikacin, 6 strains with aztreonam, piperacillin and amikacin and 4 strains with aztreonam, piperacillin/tazobactam and amikacin. Thus, the combination of aztreonam, ceftazidime and amikacin was the most effective, and therefore, the *in vitro *antimicrobial effects of this drug combination were evaluated.

Bactericidal activity was defined as a ≤ 3 log_10 _cfu/ml decrease in the starting inoculum. A bacteriostatic effect was defined as any decrease in the viable count from the starting inoculum [17].

### Data analysis

In the killing tests, the effects on the 23 strains of MBL-producing *P. aeruginosa *were compared among aztreonam alone, colistin alone and the three-drug combination of aztreonam, ceftazidime and amikacin using the Kruskal-Wallis test based on the decrease in the viable count from the initial count at 2, 4, 6 and 24 h after drug addition.

## Results

Of the 23 MBL-producing *P. aeruginosa *strains, 1 strain (strain no. 7) was *bla*_VIM-2_, and the other 22 strains were *bla*_IPM-1 _by MBL typing. All 23 strains tested were confirmed to differ in their DNA pattern by pulsed-field gel electrophoresis. Concerning differences in the PFGE pattern, a one band difference was observed in 10 strains of 5 groups, 2–3 band differences in 5 strains of 2 groups, and more than 3 band differences in the other strains. Table S1 [additional file 1] shows the MICs of the 11 drugs against the 23 strains of MBL-producing *P. aeruginosa*. The MIC of piperacillin was ≤ 64 mg/l in 16 (69.6%) of the 23 strains. The MIC of piperacillin/tazobactam was ≤ 64 mg/l in 20 (87.0%), that of aztreonam was ≤ 16 mg/l in 13 (56.5%) and that of colistin was ≤ 2 mg/l in all 23 strains. However, the MICs of the other antimicrobial agents were high in most strains.

**Figure 1:**
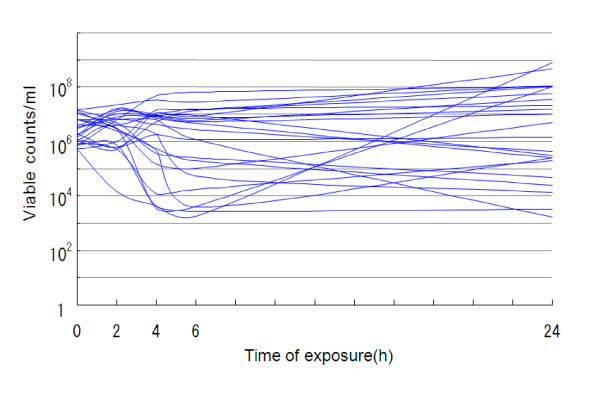
**Bactericidal effects of 48 mg/l aztreonam against 23 strains of metallo-*β*-lactamase-producing *P. aeruginosa *(37°C)**.

**Figure 2:**
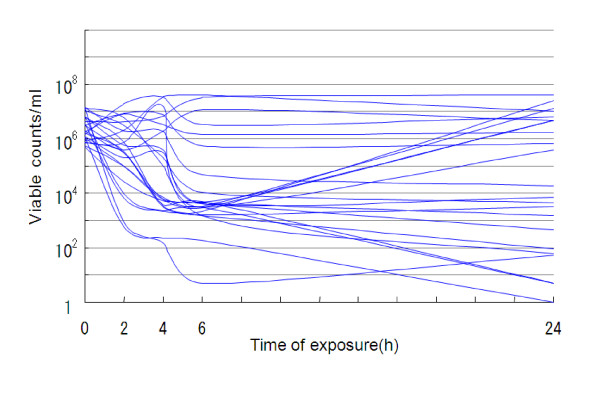
**Bactericidal effects of the 3-drug combination of 16 mg/l aztreonam, 16 mg/l ceftazidime and 4 mg/l amikacin against 23 strains of metallo-*β*-lactamase-producing *P. aeruginosa *(37°C)**.

**Figure 3:**
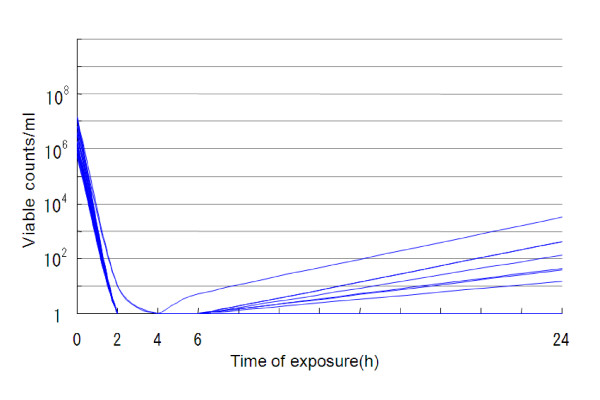
**Bactericidal effects of 2 mg/L colistin against 23 strains of metallo-*β*-lactamase-producing *P. aeruginosa *(37°C)**.

Aztreonam (48 mg/l) had bacteriostatic effects on 43.5–56.5% of the strains but bactericidal effects on only 0–4.3% at 2–24 h after its addition (Table 1, Figure 1). The 3-drug combination of aztreonam (16 mg/l), ceftazidime (16 mg/l) and amikacin (4 mg/l) had bacteriostatic effects on 69.6–82.6% of the strains and bactericidal effects on 8.7–39.1% at 2–24 h after their addition (Table 2, Figure 2). On the other hand, colistin (2 mg/l) exhibited bactericidal effects on all strains (100%) at 2–24 h after its addition (Table 3, Figure 3). Kruskal-Wallis tests showed significant decreases in the viable cell count at 2, 4, 6 and 24 h after the addition of colistin (Figure 3) compared with aztreonam alone (Figure 1) or the 3-drug combination of aztreonam, ceftazidime and amikacin (Figure 2).

**Table 1:** Antimicrobial effects of aztreonam (48 mg/l) against 23 strains of metallo-*β*-lactamase-producing *P. aeruginosa*

	No. of strains (%) showing effects^a^	
	
Time of exposure (h)	Bacteriostatic effects	Bactericidal effects
2	13 (56.5)	0 (0)
4	10 (43.5)	0 (0)
6	12 (52.2)	1 (4.3)
24	11 (47.8)	1 (4.3)

^a^Bacteriostatic effects, any decrease in the viable count from the starting inoculum; bactericidal effects, ≥ 3 log cfu/ml decrease in the starting inoculum.

**Table 2:** Antimicrobial effects of the 3-drug combination of aztreonam (16 mg/l), ceftazidime (16 mg/l) and amikacin (4 mg/l) against 23 strains of metallo-*β*-lactamase-producing *P. aeruginosa*

	No. of strains (%) showing effects^a^	
	
Time of exposure (h)	Bacteriostatic effects	Bactericidal effects
2	18 (78.3)	2 (8.7)
4	16 (69.6)	5 (21.7)
6	19 (82.6)	7 (30.4)
24	16 (69.6)	9 (39.1)

^a^Bacteriostatic effects, any decrease in the viable count from the starting inoculum; bactericidal effects, ≥ 3 log cfu/ml decrease in the starting inoculum.

**Table 3:** Antimicrobial effects of colistin (2 mg/l) against 23 strains of metallo-*β*-lactamase-producing *P. aeruginosa*

	No. of strains (%) showing effects^a^	
	
Time of exposure (h)	Bacteriostatic effects	Bactericidal effects
2	23 (100)	23 (100)
4	23 (100)	23 (100)
6	23 (100)	23 (100)
24	23 (100)	23 (100)

^a^Bacteriostatic effects, any decrease in the viable count from the starting inoculum; bactericidal effects, ≥ 3 log cfu/ml decrease in the starting inoculum.

## Discussion

No hydrolysis of aztreonam by MBL has been reported, and studies using an animal model of pneumonia infection with a VIM-2-positive *P. aeruginosa *isolate showed that a high-dose of aztreonam reduced the bacterial load and may be a useful drug [20,21]. Aztreonam is negligibly toxic and can be administered at high doses. After high-dose administration, a blood concentration three times the breakpoint can be achieved [22].

A 2-drug combination of a *β*-lactam antibiotic and an aminoglycoside antibiotic was reported to be effective against *P. aeruginosa *[16,19,23-27]. However, for multidrug-resistant *P. aeruginosa*, 3-drug combinations such as that of aztreonam, ceftazidime and amikacin rather than 2-drug combinations were shown to exhibit more marked *in vitro *antimicrobial effects [8,9]. Such observation is based on experiments in one country. In addition, colistin is effective *in vitro *against multidrug-resistant *P. aeruginosa *[28-30]. Therefore, we evaluated the bacteriostatic and bactericidal effects of aztreonam, a 3-drug combination (aztreonam, ceftazidime and amikacin) and colistin against 23 strains of MBL-producing *P. aeruginosa*. We found that although aztreonam had relatively low MIC (≤ 64 mg/l) among *β*-lactam antimicrobial agents against 21 of the 23 strains of MBL-producing *P. aeruginosa *[additional file 1] the drug at 3-fold the breakpoint concentration, 48 mg/l, had bactericidal effects only on 1 of the 23 strains when used alone (Table 1).

On the other hand, the 3-drug combination of aztreonam, ceftazidime and amikacin showed bacteriostatic effects against 19 (82.6%) of the 23 MBL-producing strains and bactericidal effects against 7 (30.4%) of the 23 strains at 6 h after drug addition, indicating relatively good *in vitro *antimicrobial effects. Therefore, the combination of the 3 drugs should be considered as a treatment method for infection with MBL-producing *P. aeruginosa*.

Colistin had more marked *in vitro *antimicrobial effects than that of the 3-drug combination against MBL-producing *P. aeruginosa*, showing bactericidal effects against all 23 strains at 2–24 h after drug addition. Although colistin has severe side effects such as renal damage [31,32], some studies showed the clinical effectiveness of colistin against multidrug-resistant *P. aeruginosa *[28,29].

## Conclusion

Evaluation of *in vitro *antimicrobial effects on metallo-*β*-lactamase-producing *P. aeruginosa *revealed relatively good effects of the 3-drug combination of aztreonam, ceftazidime and amikacin and marked effects of colistin.

## Competing interests

The authors declare that they have no competing interests.

## Authors' contributions

SO and KA conceived the idea for the study; YF, MY, and YM collected the data; SO and KA drafted the manuscript. All authors contributed in the writing and preparation of the manuscript. All authors read and approved the final manuscript.

## Pre-publication history

The pre-publication history for this paper can be accessed here:

http://www.biomedcentral.com/1471-2334/9/123/prepub

## Supplementary Material

Additional file 1 **Table S1 MICs (mg/l) of 11 drugs against 23 strains of metallo-*β*-lactamase-producing *Pseudomonas aeruginosa***. The data provided the MICs of the 11 drugs against the 23 strains of MBL-producing *P. aeruginosa*. Click here for file
