# Supplementary material for: In vitro antimicrobial effects of aztreonam, colistin, and the 3-drug combination of aztreonam, ceftazidime and amikacin on metallo-β-lactamase-producing Pseudomonas aeruginosa
Source: BMC Infect Dis. 2009 Aug 10;9:123. doi: 10.1186/1471-2334-9-123 (PMC2738676; doi:10.1186/1471-2334-9-123)
Supplement: Additional file 1 — Table S1 MICs (mg/l) of 11 drugs against 23 strains of metallo-β-lactamase-producing Pseudomonas aeruginosa. The data provided the MICs of the 11 drugs against the 23 strains of MBL-producing P. aeruginosa. [file 1471-2334-9-123-S1.doc]

Table 1. MICs (mg/l) of 11 drugs against 23 strains of metallo-*β*-lactamase-producing *Pseudomonas aeruginosa*

| Agent | MIC (mg/l) for strain no.^a^ | | | | | | | | | | | | | | | | | | | | | | | Resistant  strains(%) |
| --- | --- | --- | --- | --- | --- | --- | --- | --- | --- | --- | --- | --- | --- | --- | --- | --- | --- | --- | --- | --- | --- | --- | --- | --- |
|  | 1 | 2 | 3 | 4 | 5 | 6 | 7 | 8 | 9 | 10 | 11 | 12 | 13 | 14 | 15 | 16 | 17 | 18 | 19 | 20 | 21 | 22 | 23 |  |
| Piperacillin | 128 | 8 | 128 | 64 | 64 | 256 | 32 | 32 | 128 | 64 | 64 | >256 | 64 | 64 | 32 | 32 | 128 | 64 | 64 | 128 | 32 | 64 | 16 | 30.4 |
| Piperacillin/  tazobactam^b^ | 32 | 8 | 32 | 8 | 32 | 128 | 8 | 16 | 128 | 8 | 16 | 256 | 16 | 32 | 16 | 8 | 32 | 16 | 32 | 32 | 16 | 16 | 8 | 13.0 |
| Imipenem | 128 | 256 | 128 | 128 | 128 | 128 | >256 | >256 | >256 | >256 | 64 | 128 | 128 | 32 | 128 | >256 | 128 | 128 | >256 | 128 | 32 | 64 | 256 | 100 |
| Meropenem | 128 | >256 | >256 | 128 | 128 | 128 | >256 | >256 | 128 | >256 | >256 | >256 | 256 | >256 | >256 | >256 | 256 | 256 | 256 | >256 | >256 | 256 | 256 | 100 |
| Aztreonam | 16 | 4 | 8 | 2 | 8 | 8 | 32 | 64 | 256 | 16 | 64 | 64 | 16 | 128 | 16 | 2 | 4 | 16 | 64 | 64 | 64 | 8 | 32 | 43.5 |
| Ceftazidime | 256 | >256 | >256 | 128 | 256 | >256 | >256 | >256 | 64 | >256 | >256 | >256 | >256 | 256 | >256 | >256 | >256 | 128 | >256 | >256 | >256 | >256 | >256 | 100 |
| Amikacin | 32 | 128 | >256 | 128 | 256 | 32 | 16 | 128 | 16 | >256 | 32 | 128 | >256 | 256 | 256 | 32 | 256 | >256 | >256 | 256 | >256 | 256 | >256 | 100 |
| Tobramycin | 256 | >256 | >256 | 256 | >256 | 32 | 256 | 256 | 256 | >256 | >256 | >256 | >256 | 256 | 64 | >256 | >256 | >256 | >256 | >256 | >256 | 256 | >256 | 100 |
| Arbekacin | 64 | 64 | 128 | 64 | 128 | 64 | 64 | 32 | 32 | 128 | 128 | 256 | 128 | 32 | 4 | 128 | 128 | 128 | 128 | 64 | 256 | 32 | >256 | 100 |
| Ciprofloxacin | 16 | 128 | 128 | >256 | >256 | 32 | 256 | 256 | 128 | 128 | >256 | 256 | 256 | 256 | 128 | 64 | 32 | 64 | 64 | 128 | 256 | 64 | 8 | 100 |
| Colistin | 2 | 2 | 2 | 2 | 2 | 2 | 2 | 2 | <0.25 | 2 | 2 | 2 | 2 | 1 | 2 | 0.5 | 2 | 2 | 2 | 2 | 2 | 2 | 2 | 0 |

^a^Metallo-β-lactamase gene typing by PCR suggested that strain no. 7 carries *bla*_VIM-2_, and the other 22 strains carry *bla*_IPM-1_.

^b^MICs of piperacillin in the presence of 4 mg/l tazobactam
